# Discrete phenotypes are not underpinned by genome-wide genetic differentiation in the squat lobster *Munida gregaria* (Crustacea: Decapoda: Munididae): a multi-marker study covering the Patagonian shelf

**DOI:** 10.1186/s12862-016-0836-4

**Published:** 2016-12-01

**Authors:** Chen Wang, Shobhit Agrawal, Jürgen Laudien, Vreni Häussermann, Christoph Held

**Affiliations:** 1 Alfred Wegener Institute, Helmholtz Center for Polar- and Marine Research, Am Handelshafen 12, 27570 Bremerhaven, Germany; 2 Universidad Católica de Valparaíso, Facultad de Recursos Naturales, Escuela de Ciencias del Mar, Avda. Brasil 2950, Valparaíso, Chile; 3 Huinay Scientific Field Station, Huinay, Los Lagos Chile

**Keywords:** Phenotypic plasticity, Genetic homogeneity, Squat lobster, Microsatellites, Gene flow

## Abstract

**Background:**

DNA barcoding has demonstrated that many discrete phenotypes are in fact genetically distinct (pseudo)cryptic species. Genetically identical, isogenic individuals, however, can also express similarly different phenotypes in response to a trigger condition, e.g. in the environment. This alternative explanation to cryptic speciation often remains untested because it requires considerable effort to reject the hypothesis that the observed underlying genetic homogeneity of the different phenotypes may be trivially caused by too slowly evolving molecular markers.

The widespread squat lobster *Munida gregaria* comprises two discrete ecotypes, *gregaria* s. str. and *subrugosa*, which were long regarded as different species due to marked differences in morphological, ecological and behavioral traits. We studied the morphometry and genetics of *M. gregaria* s. l. and tested (1) whether the phenotypic differences remain stable after continental-scale sampling and inclusion of different life stages, (2) and whether each phenotype is underpinned by a specific genotype.

**Results:**

A total number of 219 *gregaria* s. str. and *subrugosa* individuals from 25 stations encompassing almost entire range in South America were included in morphological and genetic analyses using nine unlinked hypervariable microsatellites and new COI sequences. Results from the PCA and using discriminant functions demonstrated that the morphology of the two forms remains discrete. The mitochondrial data showed a shallow, star-like haplotype network and complete overlap of genetic distances within and among ecotypes. Coalescent-based species delimitation methods, PTP and GMYC, coherently suggested that haplotypes of both ecotypes forms a single species. Although all microsatellite markers possess sufficient genetic variation, AMOVA, PCoA and Bayesian clustering approaches revealed no genetic clusters corresponding to ecotypes or geographic units across the entire South-American distribution. No evidence of isolation-by-distance could be detected for this species in South America.

**Conclusions:**

Despite their pronounced bimodal morphologies and different lifestyles, the *gregaria* s. str. and *subrugosa* ecotypes form a single, dimorphic species *M. gregaria* s. l.. Based on adequate geographic coverage and multiple independent polymorphic loci, there is no indication that each phenotype may have a unique genetic basis, leaving phenotypic plasticity or localized genomic islands of speciation as possible explanations.

**Electronic supplementary material:**

The online version of this article (doi:10.1186/s12862-016-0836-4) contains supplementary material, which is available to authorized users.

## Background

Different species have different morphologies and lifestyles, which is commonly taken (but not often tested) to reflect different underlying genotypes. The advent of affordable DNA sequencing and molecular barcoding have served to greatly intensify the crosstalk between molecular and taxonomic disciplines by uncovering a large number of previously overlooked genotypes [1–3], many of which could be shown to be associated with equally overlooked morphotypes that in retrospect were identified as (pseudo)cryptic species [4–6].

However, the popularity and large number of cryptic species currently being discovered have led to an under-appreciation of the notion that sharply distinct morphotypes are not always the consequence of genetic differences but can also be invoked from the same genotype, often called by environmental triggers. The differences between associated morphotypes and lifestyles of ecotypes within the same species can be surprisingly pronounced [7–11].

Proving polyphenism and rejecting cryptic speciation as an explanation is harder than sequencing a mitochondrial gene fragment, which may in part explain the relatively lower number of well-studied cases of polyphenism. Whilst consistent differences among different morphotypes in a single mitochondrial marker suffice to at least flag these clades as candidate cryptic species, the opposite observation (no consistent differences) is not a conclusive demonstration of the absence of genetic differentiation among ecotypes. In order to show that too slowly evolving markers or other artefacts (e.g. mito-nuclear discordance [12]) did not trivially cause the observed lack of differentiation, considerably more extensive molecular evidence including multiple unlinked nuclear loci with sufficiently high substitution rates is required. Such extensive *a posteriori* knowledge is rare (e.g. in the fully sequenced *Daphnia pulex* [13–15]), but numerous experimental studies in which the genetic identity of individuals is known *a priori* contribute greatly to our understanding of the importance of polyphenism and morphological plasticity, e.g. parthenogenetic aphids [16, 17], marbled crayfish [18], polyembryonic armadillos [19], inbred lines of *Drosophila* [20], cloned swine [21]. It is unclear if the small number of confirmed polyphenism resulting from similar or identical genetic backgrounds is a condition truly rare in nature or whether it reflects mostly a discovery and/or publication bias.

**Fig. 1:**
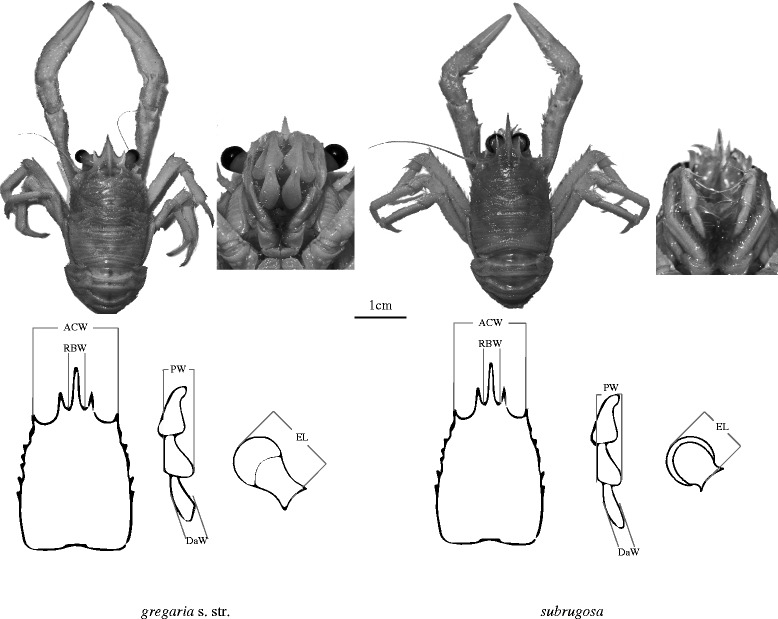
General views of *gregaria* s. str. and *subrugosa* ecotypes (above) and schematic diagrams showing morphometric measurements (below). For the dorsal view of both ecotypes, scale bar represents 1 cm. Measurements are made on: ACW, anterior carapace width; RBW, rostrum basis width; DaW, width of dactylopodus of third maxilliped; PW, width of propodus of the third maxilliped; EL, eyestalk length

In this paper we investigate the dimorphic squat lobster, *Munida gregaria* sensu lato (Fabricius, 1793), which is currently considered to comprise the ecotypes *M. gregaria* sensu stricto Miers 1881 as well as its junior synonym *M. subrugosa* Dana, 1852 (see [22]). For clarity and brevity, hereafter we refer to them as *Munida gregaria* s.l. comprising the two ecotypes *gregaria* s.str. and *subrugosa*. In South America, *M. gregaria* s. l. occurs in shallow marine waters off Patagonia, including Tierra del Fuego and the Falkland Islands/Islas Malvinas, while in the southwestern Pacific *M. gregaria* s. l. are reported from off eastern New Zealand and Tasmania ([22, 23] and references therein). The taxonomic status of *gregaria* s. str. and *subrugosa* ecotypes has been subject to conflicting interpretations. Both ecotypes were often regarded as different species because of morphological differences in adult specimen (Fig. 1) as well as different behaviors at certain developmental stages [24–27]. Williams (1973) on the other hand interpreted *gregaria* s. str. as a transient, pelagic ontogenetic stage that would later in life gain the physical features of *subrugosa* upon adopting a permanently benthic lifestyle. Regardless of the taxonomic ramifications, g*regaria* s. str. is often found in huge pelagic swarms that *subrugosa* lacks [23, 28]. These differences persist even where both ecotypes co-exist in the same habitat. Nevertheless, on the basis of a lack of mitochondrial DNA differentiation [29], these two ecotypes are currently treated as a single polymorphic species under the name of *M. gregaria* in the most recent taxonomic revision of the family [22]. But this evidence must be considered insufficient because the sampled region (Beagle Channel) represents a very small part of the species’ distribution and the molecular evidence rest exclusively on two linked mitochondrial markers (COI and ND1), whereas the results of the only nuclear marker (ITS-1) had to be excluded from the final analysis of the only molecular study [29].

In order to test whether *gregaria* s. str. and *subrugosa* ecotypes correspond to different species of *Munida* or represent a single species with variable phenotypes, we employed multiple independent, fast-evolving nuclear microsatellite markers [30] and an expanded set of mtDNA sequences. The sampled area encompasses nearly the entire distribution of *gregaria* s. str. and *subrugosa* ecotypes in South America. In addition, we analyzed morphological differences of both ecotypes and different ontogenetic stages following the method of [27] in order to test if the more complete geographic sampling continues to support the discrete morphological clusters or if the boundaries between the two ecotypes vanish under more complete geographic coverage.

## Methods

### Study population

**Fig. 2:**
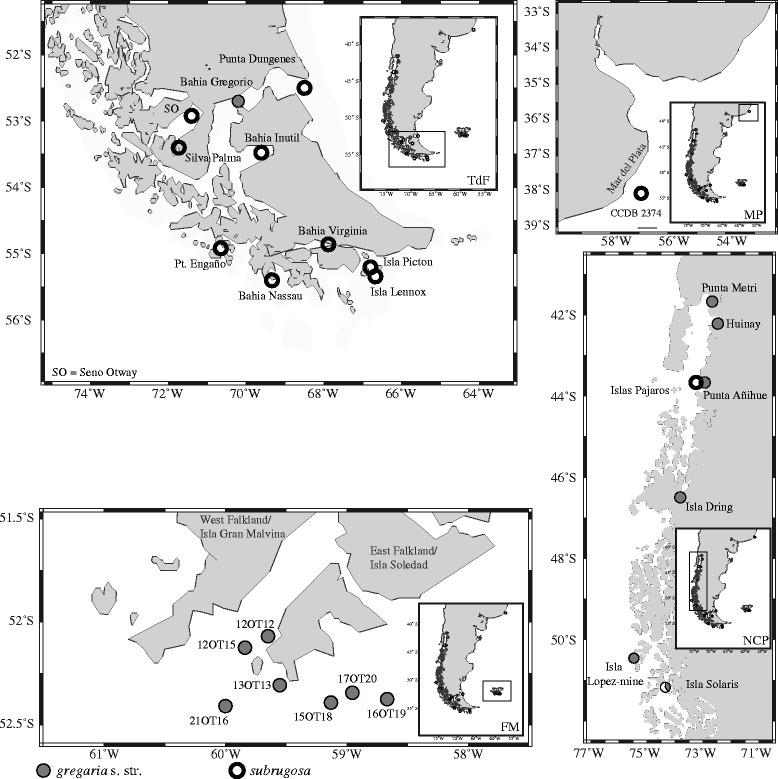
Sampling sites of *gregaria* s. str. (solid circle) and *subrugosa* (open circle) ecotypes. FM, Falklands/Malvinas; TdF, Tierra del Fuego archipelago; NCP, Northern Chilean Patagonia; MP, Mar del Plata

A total number of 219 individuals were used for both morphological and molecular analyses in this study. These samples were collected at 25 stations from Patagonia and off the Falkland Islands/Islas Malvinas ranging from 38° S to 55° S from the shallow subtidal area to 179 m water depth by mid-water or bottom trawls (Table 1, Fig. 2). Sexual maturity was identified as presence of eggs and/or sexually dimorphic pleopods. In adult males the first two pairs of pleopods are modified to form gonopods, the remaining three pairs are flap-like; in adult females the first pair is missing and the remaining four pairs are elongated with long setae for egg-carrying [28].

**Table 1:** Sampling sites and number of adult and juvenile (in parentheses) *gregaria* s. str. and *subrugosa* ecotypes

Station	Latitude	Longitude	*gregaria* s. str. adults (juveniles)	*subrugosa* adults (juveniles)
			*N*_*mtDNA*_/*N*_*MSAT*_	*N*_*mtDNA*_/*N*_*MSAT*_
Falklands/Malvinas (FM)				
12OT12	−52.110	−59.595	3/16 (0/0)	0/0 (0/0)
13OT13	−52.285	−59.546	4/12 (0/0)	0/0 (0/0)
15OT18	−52.391	−59.131	2/16 (0/0)	0/0 (0/0)
16OT19	−52.354	−58.592	2/16 (0/0)	0/0 (0/0)
17OT20	−52.359	−58.889	2/16^a^ (0/0)	0/0 (0/0)
21OT16	−52.408	−59.976	2/16^a^ (0/0)	0/0 (0/0)
12OT15	−52.189	−59.975	1/1 (0/0)	0/0 (0/0)
Total			16/93 (0/0)	0/0 (0/0)
Northern Chilean Patagonia (NCP)				
Punta Metri	−41.595	−72.712	0/0 (4/7)	0/0 (0/0)
Huinay	−42.354	−72.463	3/3 (13/13)	0/0 (0/0)
Isla Dring	−46.442	−73.957	0/0 (7/7)	0/0 (0/0)
Punta Añihue	−43.793	−72.925	0/0 (4/16)	0/0 (0/0)
Islas Pajaros	−43.783	−72.997	0/0 (0/0)	0/0 (5/5)
Isla Lopez-mine	−50.361	−75.332	0/0 (4/7)	0/0 (0/0)
Isla Solaris	−51.330	−74.311	5/5 (0/0)	7/7 (0/0)
Total			8/8 (32/50)	7/7 (5/5)
Tierra del Fuego archipelago (TdF)				
Punta Dungenes	−52.436	−68.568	0/0 (0/0)	1/1 (0/0)
Bahia Gregorio	−52.685	−70.142	5/10 (0/0)	0/0 (0/0)
Seno Otway	−52.918	−71.347	0/0 (0/0)	4/13 (0/0)
Silva Palma	−53.347	−71.800	0/0 (0/0)	0/0 (2/7)
Bahia Inutil	−53.556	−69.695	0/0 (0/0)	2/5 (0/0)
Bahia Nassau	−55.383	−69.450	0/0 (0/0)	1/1 (0/0)
Pt. Engaño	−54.929	−70.709	0/0 (0/0)	2/2 (0/0)
Bahia Virginia	−54.911	−67.726	0/0 (0/0)	8/12 (0/0)
Isla Picton	−55.174	−66.721	0/0 (0/0)	0/2 (0/0)
Isla Lennox	−55.393	−66.679	0/0 (0/0)	2/2 (0/0)
Total			5/10 (0/0)	20/38 (2/7)
Mar del Plata (MdP)				
CCDB 2374	−38.003	−57.469	0/0 (0/0)	1/0 (0/0)
Grand total			29/111 (32/50)	28/45 (7/12)

*N*_*mtDNA*_ the number of specimens used with mitochondrial marker, *N*_*MSAT*_ the number of specimens used with microsatellites

^a^refers to one specimen with missing genotype at a certain microsatellite locus

### Morphological analysis

Specimens were checked by eye and classified as *gregaria* s. str. rather than *subrugosa* ecotypes based on the following characteristics: longer eyestalk length (EL), wider rostrum basis (RBW) and broader and blunter dactylus of the third maxilliped (DaW) (Fig. 1). These three morphometric characteristics together with anterior carapace width (ACW) and width of propodus of the third maxilliped (PW) were statistically significant in discriminating ecotypes [27]. We measured our samples using a Leica MZ-12.5 microscope with intraocular scale to the nearest 0.1 mm. To determine patterns emerging from the morphometric measurements of these five body parts, principal components analysis (PCA) was plotted using the statistical package PAST 3 (PAlaeontological STatistics, [31]). Applying discriminant functions (DF1 and DF2) introduced in [27], ΔDF (DF1-DF2) values were calculated based on these measurements and subsequently plotted using R [32].

### mtDNA analysis

DNA was extracted from ethanol-preserved abdominal or cheliped muscle tissue using QIAamp DNA Mini Kit (QIAGEN, Germany). For mtDNA analysis, a region of the COI gene was amplified using the universal primers HCO2198 and LCO1490 [33] for 96 individuals (Table 1). The 10 μl reactions consisted of 0.02U/μl Hotmaster Taq (5 Prime), 0.2 mM dNTPs, 0.5 μM of forward and reverse primers, 1 × PCR-buffer and 1 μl (about 30 ng) of template DNA. PCR was conducted using an initial denaturation at 94 °C for 2 min, followed by 36 cycles of 94 °C for 20 s, annealing at 47 °C for 20 s, 65 °C for 1 min, and a final extension at 65 °C for 10 min. Size and quality of amplified products were checked on a 2% agarose gel in TAE buffer, and then 1 μl of purified PCR product was used for cycle sequencing with the HCO primer. Sanger sequencing was conducted on an ABI 3130xl sequencer. Alignment was done using CODONCODE ALIGNER 4.0 (CodonCode Corp.) and checked for the presence of ambiguities and stop codons.

DNA polymorphism was examined as haplotype diversity (H_D_) and nucleotide diversity (π) for each ecotype and all samples using DnaSP 5.10 [34]. Genealogical relationships among haplotypes were inferred using statistical parsimony implemented in TCS 1.21 [35]. Pairwise genetic divergences measured as number of nucleotide differences were calculated within and between the two ecotypes in MEGA 5.2 [36]. For a better understanding of the genetic distances and barcoding gap analysis, we added three congeneric species to our analysis: *M. rutllanti* (*n* = 5; GenBank accession numbers: JQ306226-JQ306230), *M. quadrispina* (*n* = 3; GenBank accession numbers: DQ882090-DQ882092), and *M. gracilis* from our own collection (*n* = 2; GenBank accession numbers: KJ544249-KJ544250). Pairwise genetic distances were calculated within *M. gregaria* s. l. (pooled *gregaria* s. str. and *subrugosa*) and versus the other three *Munida* species.

The COI dataset was analyzed using coalescent based approaches Poisson tree processes (PTP) model [37] and the general mixed Yule coalescent model (GMYC) [38, 39] for a critical evaluation of species delimitation. As an outgroup, COI sequences from four congeners, *M. rutllanti* (*n* = 5; GenBank accession numbers: JQ306226-JQ306230), *M. quadrispina* (*n* = 2; GenBank accession numbers: DQ882090 and DQ882092), *M. rosula* (*n* = 1; GenBank accession numbers: AY350994) and *M. congesta* (*n* = 1; GenBank accession numbers: AY350945), were added. The substitution model that best fits the data was determined using jModelTest 2.1.5 [40, 41].

PTP does not require an ultrametric tree, as the transition point between intra- and inter-specific branching rates is identified using directly the number of nucleotide substitution [37]. A maximum likelihood (ML) phylogeny of the COI dataset was reconstructed in RAxML-HPC 8 in CIPRES portal [42, 43], employing a HKY + G model that was suggested by the corrected Akaike Information Criterion (AICc) and the Bayesian Information Criterion (BIC). Nodal support was evaluated using 1000 bootstrap replicates. The ML phylogenetic tree was used as the input tree to run PTP species delimitation analysis in the PTP webserver (http://species.h-its.org/ptp/). We ran the PTP analysis for 500,000 MCMC generations, with a thinning value of 100 a burn-in of 10%. Outgroup taxa were kept since the MCMC chains did not converge when they were removed.

The GMYC method requires a fully resolved tree with branch lengths estimates, which was obtained using the program BEAST 2.4.3 [44]. We used a site-specific HKY substitution matrix and a gamma distributed model of among-site rate heterogeneity with four discrete rate categories. We implemented a strict clock model of 2% per Myr as suggested for COI sequences in Crustacea [45, 46], and selected a Yule tree prior. Default values were used for remaining priors. MCMC analysis was run for a total of 10 million generations, sampling every 1000 steps. Convergence was assessed by examining the likelihood plots through time using TRACER 1.6 [47]. The COI chronogram was then analyzed using the GMYC package in SPLITS in R (version 3.1.2, www.cran.r-project.org), using the single threshold approach [38, 39].

### Microsatellite analysis

In total, 218 individuals were screened for genetic variation at 11 microsatellite loci that were originally designed for *M. gregaria* s. l. [30] (Table 1). Allele sizes were binned manually and genotypes were assessed in GENEMAPPER 4.0 (Applied Biosystems). Null alleles, stuttering and large allele dropout were tested using MICROCHECKER [48]. Because of too many missing data and possible null alleles, locus Mgr63 and Mgr105 were excluded from subsequent analyses. Genetic diversity within each ecotype was summarized as allelic richness (*Ar*) in FSTAT 2.9.3.2 [49] using the rarefaction approach, which was also used to determine the number of private alleles using standardized sample sizes in ADZE 1.0 [50]. Detection of linkage disequilibrium between loci and deviations from Hardy-Weinberg equilibrium (HWE) per ecotype were performed using GENEPOP 4.2 [51]. All loci were tested for positive/diversifying or balancing selection using LOSITAN [52], which simulates an expected distribution of *F*_ST_ as a function of expected heterozygosity under an island model of migration. The statistical power of this set of microsatellite loci to detect significant genetic differentiation between populations/ecotypes was tested with POWSIM 4.1 [53] using both Chi-square (*χ* ^2^) and Fisher’s exact test analysis. Various levels of differentiation (measured as *F*_ST_ in the range from 0.001 to 0.01) were determined by combining different effective population size (*N*_*e*_) and times since divergence (*t*). In addition, POWSIM allows calculating type I error probability, which is the probability of rejecting the null hypothesis of genetic homogeneity although it was true by drawing the alleles directly from the base population (*t* = 0).

The genetic differentiation among the three major sampling areas, i.e., FM, NCP and TdF (see Table 1), was assessed for each ecotype separately with AMOVA in ARLEQUIN 3.5 [54]. Both *F*_ST_ and *R*_ST_ estimators were calculated over all nine loci with 1000 permutations. To provide a visual representation of species separation and potential subdivision, Principal Coordinate Analysis (PCoA) was performed in GENALEX 6.5 [55].

Bayesian assignment tests were used to evaluate the level of genetic clustering. We used STRUCTURE 2.3.4 [56] first without giving any prior population information, letting *K* range from 1 to 5. We also checked whether individuals could be assigned correctly to clusters if the number of ecotypes was given *a priori* (*K* = 2). Both conditions were run with the correlated allele frequencies option under the non-admixture model, i.e. under the assumption that there is no gene flow between ecotypes, as well as under an admixture model, i.e. allowing limited introgression between clusters. Twenty runs with 200,000 Markov chain Monte Carlo (MCMC) iterations after a burn-in period of 25,000 steps were carried out for each *K*. The results were uploaded onto STRUCTURE HARVESTER [57] and *K* was determined using the *ad hoc* statistic Δ*K* [58], as well as mean estimates of posterior probability *L(K)* [56]. Results from the 20 replicates of the most likely value for *K* were averaged using the software CLUMPP 1.1.2 [59] and the output was visualized using DISTRUCT 1.1 [60].

Since *L(K)* does not always provide the correct number of clusters and the Δ*K* statistic cannot evaluate *K* = 1 or the largest value chosen for *K* [58], we also applied STRUCTURAMA 2.0 [61], which can directly estimate the number of clusters in which a sample can be subdivided. We allowed the number of populations to be a random variable following a Dirichlet process prior, ran the MCMC analysis for 1,000,000 cycles, sampled every 100th cycle, and discarded the first 400 samples as burn-in.

The impact of isolation by distance (IBD) across southern South America on genetic differentiation was estimated by Mantel tests as implemented in IBD Web Service 3.23 [62]. For this purpose, only the geographic position but not the ecotype of the samples (mitochondrial and microsatellite data) were used (Table 1; Fig. 2). Pairwise *F*_ST_ values for mitochondrial data and pairwise (*δμ*)^2^ genetic distance [63] for microsatellites were obtained in ARLEQUIN 3.5, where spatial distances were calculated using the Geographic Distance Matrix Generator 1.2.3 (http://biodiversityinformatics.amnh.org/open_source/gdmg/index.php). Geographical distances were log-transformed to account for two-dimensional habitat distribution [64], and the significance of the slope of the reduced major axis (RMA) regression was assessed by 30,000 randomizations.

## Results

### Morphological analysis

**Fig. 3:**
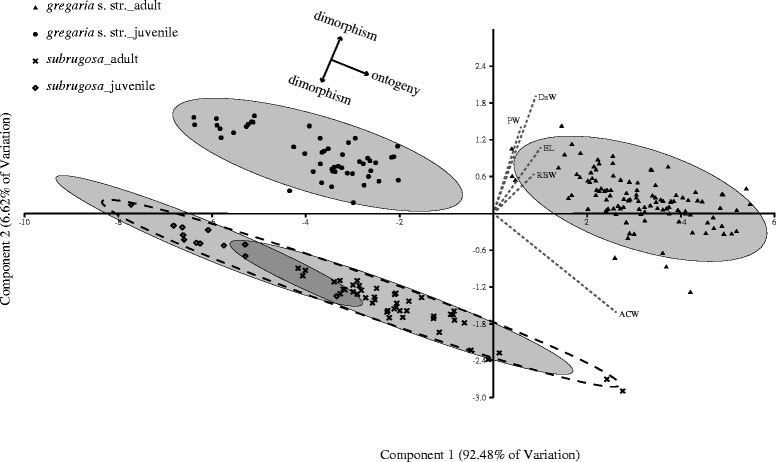
Principal component analysis (PCA) biplot for the morphometric measurements of all the samples. Dashed lines show vectors from the five representative body characteristics that are statistically significant in discriminating ecotypes. The 95% concentration ellipses are given for juvenile and adult *gregaria* s. str. as well as juvenile and adult *subrugosa*. A dashed 95% concentration ellipse represents all *subrugosa* individuals. Solid arrows indicate suggested interpretation as ontogenetic transition within ecotype (horizontal) and morphological discreteness between ecotypes (vertical; see discussion)

**Fig. 4:**
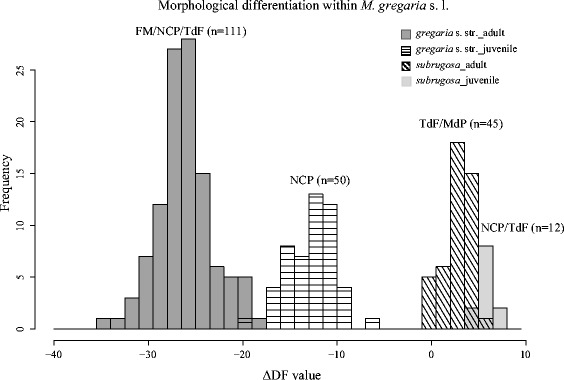
Frequency distribution of ΔDF scores based on the morphometric measurements

PCA comparison of five key morphometric characteristics [27] revealed clearly distinct groups corresponding to ecotype and age, with the first principal component explaining 92.48% of the variation. Samples of adult and juvenile *gregaria* s. str. formed two isolated groups, both of which were clearly distinct from the *subrugosa* samples. The *subrugosa* individuals comprise the adult and juvenile sub-groups that overlap in part (Fig. 3). The discriminant functions with these five morphometric characteristics yielded result coherent with the PCA. Samples of adult *gregaria* s. str. and juvenile *gregaria* s. str. clustered separately, while due to the limited number of *subrugosa* juveniles (*n* = 12) it is hard to ascertain whether juvenile and adult *subrugosa* show statistically significant differences (Fig. 4). Results of morphological analyses ascertain that even among samples from the entire South American distribution *gregaria* s. str. and *subrugosa* are distinct ecotypes at different ontogenetic stages with discrete morphological traits rather than forming the extremes of a continuous distribution.

### Mitochondrial COI sequence variation

A total number of 96 COI sequences from 61 *gregaria* s. str. and 35 *subrugosa* individuals were obtained as an alignment of 618 bp (GenBank accession numbers: KJ544251 - KJ544346). These sequences were collapsed into 30 different haplotypes, possessing 29 variable (segregating) sites, of which 11 were parsimony-informative. The *subrugosa* ecotype showed slightly higher genetic diversity than *gregaria* s. str. (Additional file 1: Table S1). In the 206 codons of the alignment, the 29 variable sites were all synonymous substitutions and no stop codons were found.

Genealogical relationships among haplotypes showed a very shallow, star-like structure. The most common haplotype (*n* = 59) was shared by both ecotypes as well as by all the sampling regions, which differed from the other haplotypes in 1 to 3 mutational steps (Additional file 2: Figure S1).

### Extent of intraspecific and interspecific COI divergence

**Fig. 5:**
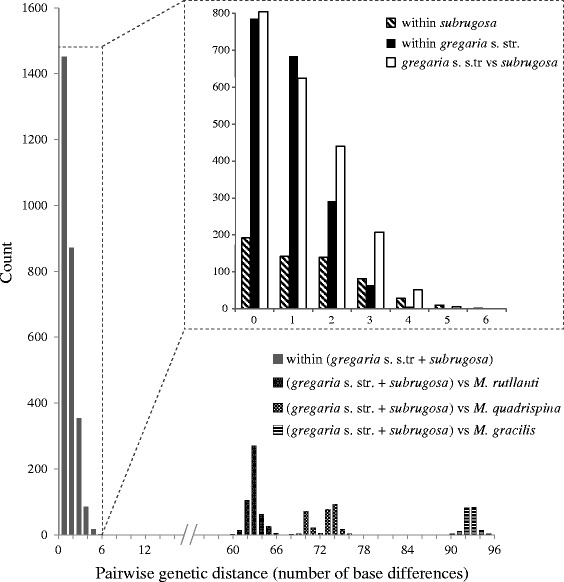
Estimates of divergence between sequences among the two ecotypes and three other *Munida* species. Pairwise comparisons were performed using number of base differences

The mean number of differences among sequences within each ecotype was 0.809 for *gregaria* s. str. and 1.408 for *subrugosa*, between ecotypes it was 1.107. The plotted pairwise genetic distances show complete overlap of distributions within and between ecotypes (Fig. 5). The maximal number of differences was six base pairs and no barcoding gap between ecotypes could be identified. By contrast, pronounced barcoding gaps exist between *M. gregaria* s. l. and each of the three other *Munida* species. These interspecific distances were at least ten times larger than distances between *gregaria* s. str. and *subrugosa* ecotypes (Fig. 5). The results of COI data including samples from almost the entire South American distribution provide no evidence of genetic separation between *gregaria* s. str. and *subrugosa* ecotypes.

### PTP and GMYC species delimitation

**Fig. 6:**
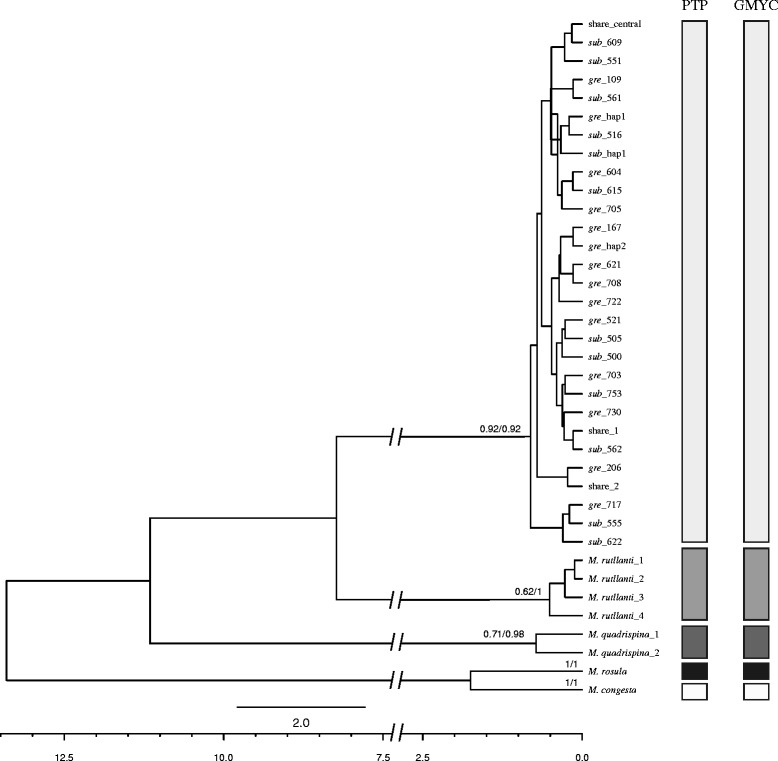
Phylogenetic relationships for *M. gregaria* s. l. and outgroup taxa. Ultrametric phylogenetic tree inferred from COI in BEAST species. Scale axis showed ages in millions of years (Ma). Species delimitation scenarios obtained from different methods are indicated in columns to the right. Numbers at nodes/tips are Bayesian support values in PTP model and AIC-based support values in GMYC model for the delimited species

The ultrametric tree obtained in BEAST was used to illustrate the delimitation of putative species recognized by the different approaches conducted with the COI data (Fig. 6). Both PTP and GMYC analyses detected 5 candidate species corresponding to the current taxonomic units, that is, the four outgroup *Munida* species and a single species *M. gregaria* including all the sequences of *gregaria* s. str. and *subrugosa*.

### Microsatellite diversity

Overall, the number of alleles per locus ranged between six (Mgr46) and 38 (Mgr60) with an average of 14.9. For each locus the number of alleles, allelic size range, allelic richness as well as observed and expected heterozygosities per population and ecotype are reported (Additional file 3: Table S2). The mean number of private alleles per locus when sample size was standardized was slightly higher for *subrugosa* (0.198 ± 0.120) compared to *gregaria* s. str. (0.111 ± 0.065), both of which were very low, reflecting a high degree of allelic sharing between the two ecotypes. Compared to the limited variation among COI sequences, the nine microsatellite loci exhibited broader allelic ranges and orders of magnitude higher allelic variation (Additional file 4: Figure S2). All loci showed no linkage disequilibrium. Locus Mgr90 showed significantly higher heterozygosity than expected, but since excluding Mgr90 had only minor effect on the results, it was kept in this study. The power test suggested that our microsatellite dataset was sensitive enough to detect very weak genetic differentiation (*F*_ST_ = 0.005) in probabilities of close to 100% using both chi-square (*χ* ^2^) and Fisher’s exact test (Additional file 5: Figure S3). The *F*_ST_ outlier analysis showed that none of the loci was under potential selection at 95% confidence level thus they were regarded as neutral in our interpretation of the results.

### Genetic differentiation and individual assignment inferred by microsatellites

**Fig. 7:**
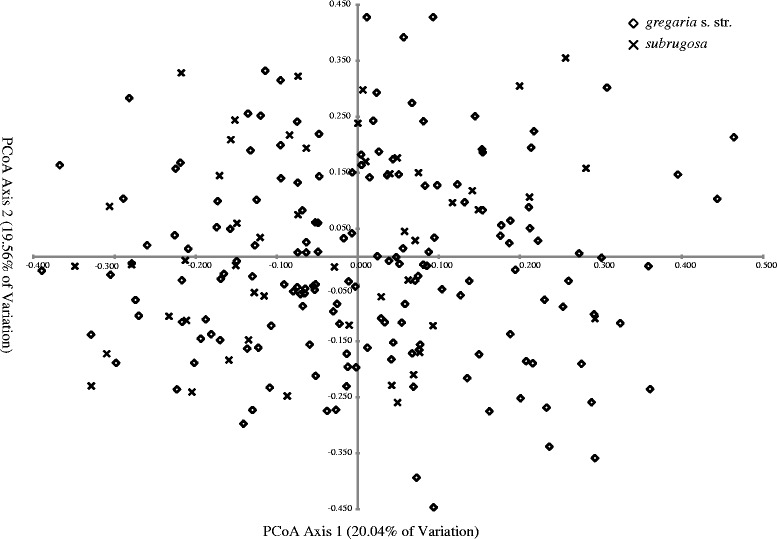
Principal coordinate analysis (PCoA) of 218 individuals of *gregaria* s. str. and *subrugosa* ecotypes based on microsatellites

Hierarchical AMOVA showed almost all the genetic variance distributed among individuals within sampling areas (Table 2), thus corroborating the weak geographic structure in the distribution of mitochondrial haplotypes. PCoA showed approximately equal distributions along the first three axes, which accounted for 20.04, 19.56 and 18.76% of the total genetic variance, respectively (Fig. 7). This result indicates that there is no single factor (ecotype or other) that would dominate the distribution of total genetic variance for the high-resolution microsatellite data.

**Table 2:** Hierarchical analysis of molecular variance based on microsatellites for both ecotypes

Source of variation	d.f.	Sum of squares	Variation components	Variation [%]	*F*-statistics	*P*	Sum of squares	Variation components	Variation [%]	*R*-statistics	*P*
Among ecotypes	1	3.487	−0.00290	−0.10	*F*_CT_ = −0.00103	0.507	167.743	−1.10788	−0.45	*R*_CT_ = −0.00448	1.00
Among sampling areas within ecotypes	3	10.977	0.01229	0.44	*F*_SC_ = 0.00437	0.0426	976.391	1.11995	0.45	*R*_SC_ = 0.00451	0.204
Within sampling areas	431	1207.396	2.80138	99.67	*F*_ST_ = 0.0334	0.0255	106578.987	247.28303	100	*R*_ST_ = 0.00005	0.277
Total	435	1221.860	2.81077	100			107723.122	247.29510	100

**Fig. 8:**
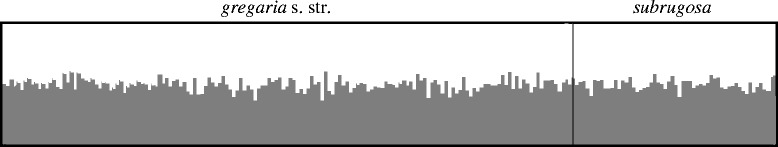
Individual probabilities of cluster assignments from the software STRUCTURE. The most likely number of clusters *K* = 2 is shown for *gregaria* s. str. (*n* = 161) and *subrugosa* (*n* = 57) using nine microsatellite loci. Each vertical line represents the probabilities for a single individual to be assigned to one of the clusters (*K*)

Bayesian cluster analyses with STRUCTURE suggested the best *K* was 1 according to average log probability (*L(K)*), but *K* = 2 was indicated by the highest statistic Δ*K*. This is because the change in log probability does not account for the smallest and largest *K*. Even under *K* = 2, each individual possessed a roughly equal probability of being assigned to the first versus the second cluster, which indicates that all individuals belong to one single group (Fig. 8). Congruent distribution of posterior probabilities for each individual was obtained given the *a priori* assumed number of putative populations (=ecotypes).

The STRUCTURAMA analysis corroborated the inferences of the STRUCTURE analysis. The sampled individuals all belonged to one group with a posterior probability of 1. Eventually, both Bayesian analyses showed no correlation of our microsatellite data with either ecotypes or geographical units.

An absence of genetic structure over the entire distribution range in South America was also found in the IBD tests. Based on both mitochondrial and microsatellite data sets, Mantel tests indicated no significant correlation (for COI, *r* = 0.0608, *P* = 0.229; for microsatellites, *r* = −0.005, *P* = 0.508) between genetic and log-transformed geographic distances (Additional file 6: Figure S4).

## Discussion

### Stability of morphological dimorphism in *M. gregaria* s. l.

In theory, populations belonging to a so-called ‘ring species’ might appear sharply distinct in an area of secondary overlap, but appear more gradually changing in morphology or genetics through areas of their distribution that have been more continuously inhabited (see [65, 66] and references therein). Inadvertently sampling only in the zone of secondary overlap might therefore create the incorrect impression of discrete morphotypes or genotypes when populations with intermediary morphotypes remain unsampled.

Increasing the sampling area from a single location in the Beagle Channel [29] to a continental scale, our data suggest that the boundary between two morphotypes (*gregaria* s. str. and *subrugosa*) is nonetheless not blurred across the South American shelf (Figs. 3 and 4).

The expanded morphometric analysis further suggests an ontogenetic dimension in the morphometry. It may be expected that the gap between adults and Northern Chilean Patagonia (NCP) juveniles in *gregaria* s. str. might be closed by inclusion of juveniles from other populations and reveal a continuous ontogenetic transition as can already be found in *subrugosa* (Fig. 3). The discreteness of the *subrugosa* and *gregaria* s. str. morphotypes, however, is not a sampling artefact and stable with respect to a more representative sampling scheme as well as inclusion of different life stages.

### Phenotype-genotype relationship

Since the proposal that phenotype and genotype form two fundamental different levels of biological abstractions [67], untangling the relationship between phenotypes and the underlying genotypes has long been challenging and intriguing. The advent of molecular techniques has greatly fostered studies of phenotype-genotype interaction, especially in the wake of helped discovery of (pseudo)cryptic genetic divergence whereas corresponding phenotypes appeared identical. Such unexpected genetic diversity, which was later often corroborated by other independent evidences from morphology [68], breeding behavior [69] or multiple, independent and informative nuclear markers [70], has become an important supplement for the phenotypic identification of an organism to species or sub-species level in taxonomic practice [71–73].

In other cases, however, molecular marker-based examination found no genetic differentiation matching discrete phenotypes, which is exemplified by the present *Munida gregaria* case. Nonetheless, the lack of differentiation at a single marker is insufficient to extrapolate to the entire genome, especially in view of the different inheritance in the mitochondrial and nuclear genomes [74, 75]. A previous molecular study used only mitochondrial evidence and found no consistent genetic differentiation associated with each ecotype [29] but failed to demonstrate genetic homogeneity in the nuclear genome. The only nuclear locus (ITS 1) was excluded from the final analysis in [29] due to conflicting information and possible paralogy of sequences. The inference of genetic homogeneity in [29] thus rested exclusively on two fully linked mitochondrial markers, COI and ND1 (the third mitochondrial marker 16S yielded identical sequences among all individuals). In the absence of recombination, mitochondrial genes are vulnerable to introgressive hybridization, sex-biased dispersal, incomplete lineage sorting and heteroplasmy [12, 76–79]. The determination of a ‘barcoding gap’ (i.e., significant difference between inter- and intraspecific variation) may fail in case of close phylogenetic relationship or recent divergence [80–82].

However, the shortcomings of previous analyses [29] were addressed by our more expansive sampling and the inclusion of multiple unlinked microsatellites, thus suggesting that the distinct phenotypes in *M. gregaria* s. l. are not caused by different genotypes.

### A case of phenotypic plasticity

A common caveat to marker-based population genetic studies in case of no differentiation detected among populations (i.e., different phenotypes in this case) is that there may be still unsampled isolated regions of differentiation within genome. Such ‘genomic islands of differentiation’ [83, 84] are usually associated with genes under divergent selection, whilst selectively neutral markers are not involved [85–87]. This alternative is hard to falsify and might be true for any marker-based study in organisms with incompletely known genomes. Adaptive divergence associated with certain selected genes has been demonstrated in the presence of gene flow [88–90]. The availability of genome-wide sequencing may help identify such individual genes, if they exist indeed, contributing to the phenotypic differentiation between the two ecotypes.

Except for the possibility of ‘genomic islands’ underpinning different phenotypes, the different ecotypes within *M. gregaria* s. l. are then strongly suggestive of phenotypic plasticity. The exact nature of a trigger that determines which of the morphotypes will be expressed is unknown at present. In similar examples from parthenogenetic *Daphnia* and aphids, sharply distinct morphotypes arise from the same genetic background [16, 17, 91] and in some examples the environmental triggers controlling which phenotype is preferentially expressed are known. The sex of offspring from the same clutch was found to be determined by temperature among various gonochoristic organisms (those having separate sexes), e.g. in invertebrates [92, 93], fishes [7, 94, 95], turtles [96] and crocodilians [97]. Dramatically different morphologies can be expressed in presence or absence of predators in *Daphnia* water fleas [98–100], barnacle *Chthamalus fissus* [9], whereas little genetic correspondence is involved in the predator-induced morphological changes [13, 14, 101].

Although genomic islands of speciation cannot be completely ruled out, some anecdotal evidence suggests that one or several as yet unknown environmental factors may be involved in the determination of *Munida* ecotypes. In its South American distribution, *Munida gregaria* s. l. occupies extensive latitudinal distribution along both coasts of Patagonia and wide bathymetry (from water surface down to 1137 m recorded for *subrugosa* [102, 103]), which involves a strong gradient of environmental conditions (temperature, salinity, oxygen concentration and food resources). In some species the feeding performance and diet composition during larval phases can induce development into different morphotypes or sex reversal [104, 105]. Since *gregaria* s. str. and *subrugosa* differ in feeding habit as deposit feeders and actively swimming planktonic feeder, respectively [106], changes in environmental food composition may affect the metamorphosis of *M. gregaria* s. l. in an adaptive fashion, favouring its development into one ecotype rather than the other. Long-term observations of the proportion of both *gregaria* s. str. and *subrugosa* ecotypes in the Beagle Channel and San Jorge Gulf demonstrate the existence of ecotypes is patchy and not stable over time (see [107] and references therein). Recent hydroacoustical evidence postulates that major pelagic swarms of *gregaria* s. str. on the Argentine continental shelf are associated with productive areas such as frontal zones that vary considerably in spatial and temporal scales [107], implying the availability of phytoplankton in frontal zones might favor the expression of *gregaria* s. str. ecotype.

Heterochrony, which is generally defined as a developmental change in relation to size and shape in the timing or rate of ontogenic events (see review in [108]), might be a possible mechanism involved in the observed plasticity in *M. gregaria* s. l.. Heterochronic process such as paedomorphic plasticity was postulated in a widespread squat lobster in the Pacific of South America, *Pleuroncodes monodon* [109]. A clear boundary exists in its distribution where to the north it is a smaller, pelagic form and to the south it is a larger, benthic form. Like *gregaria* s. str. and *subrugosa*, these two forms showed no mitochondrial DNA differentiation either. A similar developmental variation might be involved in *M. gregaria* s. l., since the population from San Jorge Gulf was shown to have faster growth rate and earlier reproductive investment in its early life history than the southern populations from Beagle Channel and Strait of Magellan [110].

Whether or not the two ecotypes spring from a genetically entirely homogenous background or whether small localized genomic islands associated with each exist, our data have made it abundantly clear that the simple, perhaps too simple, model of a genome-wide 1:1 relationship between the genotype and an associated phenotype (however ill equipped we may be to recognize the latter) does not apply to the *Munida gregaria* case.

A next-generation sequencing approach [111] with higher number of loci and vastly improved coverage of the genome is a promising way to determine if islands of genetic differentiation associated with the ecotypes are involved or if the trigger determining the expression of one or the other morphotype from identical genotypes may be independent of genetic differentiation and under the control of an extrinsic factor.

## Conclusions

Based on extensive sampling of the species’ distribution in South America and using nine independent polymorphic nuclear microsatellite loci in addition to new mitochondrial COI sequences, we were able to show that the lack of genetic differentiation between distinct *gregaria s. str.* and *subrugosa* ecotypes is not an artefact due to insufficient genomic and/or geographic sampling or slowly evolving markers. Instead they are likely expressed from a single underlying genotype although two largely identical genotypes with interspersed localized genomic islands of differentiation cannot be fully ruled out without a more complete coverage of the genome. Morphological tests affirmed the boundaries between the two ecotypes were not blurred with continental-scale geographic sampling, and remain stable despite an ontogenetic dimension in the data. These findings corroborate the current taxonomic view of *M. gregaria* s. l. (Fabricius, 1793) as a single, dimorphic species, thus demonstrating a pattern very unlike cryptic speciation commonly found in DNA taxonomy and DNA barcoding studies. Our study also emphasizes the necessity of incorporating complementary nuclear multi-locus markers in studies aiming at taxonomy and genotype-phenotype relationship, in view of the increasing numbers of reported discordance between mtDNA and nuclear DNA. *M. gregaria* is developing into a model affording deeper insights into the phenotype-genotype relationship, environmental control of ontogeny and ultimately into the process of speciation itself.

## Additional files

Additional file 1: Table S1. Genetic diversity of COI sequences per ecotype. (DOCX 15 kb)
Additional file 2: Figure S1. Haplotype genealogies for *gregaria* s. str. and *subrugosa* ecotypes. Each branch represents one substitution; small filled circles represent hypothetical, unsampled haplotypes. Radii reflect number of individuals that share a particular haplotype. FM, Falklands/Malvinas; NCP, Northern Chilean Patagonia; TdF, Tierra del Fuego archipelago; MdP, Mar del Plata. (EPS 450 kb)
Additional file 3: Table S2. Diversity indices of nine microsatellite loci for the two ecotypes. Reported are number of alleles nA, fragment size range, observed heterozygosity *H*_*O*_, expected heterozygosity *H*_*E*_ and allelic richness *Ar*. Significant deviation from Hardy-Weinberg equilibruim (*P* < 0.05, based on 10,000 permutations) after Bonferroni correction were labeled in bold. (DOCX 19 kb)
Additional file 4: Figure S2. Allelic frequencies (in logarithm) of the nine microsatellite loci polymorphism. (EPS 1291 kb)
Additional file 5: Figure S3. Tests of statistical power for microsatellite data set as inferred with POWSIM 4.1. (EPS 1184 kb)
Additional file 6: Figure S4. Correlation between genetic distances (Pairwise *F*_ST_ values for COI and pairwise (*δμ*)^2^ values for microsatellites) and log-transformed geographical distances for mitochondrial and microsatellite data for specimens from 25 sampling sites listed in Table 1. (EPS 1500 kb)
Additional file 7: Data S1. Cytochrome c oxidase subunit I (COI) sequence alignment file. (NEXUS 59 kb)
Additional file 8: Data S2. Microsatellite data file for each ecotype under three major sampling areas. (TXT 17 kb)
