# Supplementary material for: Discrete phenotypes are not underpinned by genome-wide genetic differentiation in the squat lobster Munida gregaria (Crustacea: Decapoda: Munididae): a multi-marker study covering the Patagonian shelf
Source: BMC Evol Biol. 2016 Dec 1;16:258. doi: 10.1186/s12862-016-0836-4 (PMC5131467; doi:10.1186/s12862-016-0836-4)
Supplement: Additional file 1: Table S1. — Genetic diversity of COI sequences per ecotype. (DOCX 15 kb) [file 12862_2016_836_MOESM1_ESM.docx]

Table S1 Genetic diversity of COI sequences per ecotype

|  | Sequences | Haplotypes | Private Haplotypes | H_D_* | π* |
| --- | --- | --- | --- | --- | --- |
| *gregaria* s. str. | 61 | 18 | 15 | 0.571 | 0.00131 |
| *subrugosa* | 35 | 14 | 12 | 0.677 | 0.00228 |
| overall | 96 | 30 |  | 0.622 | 0.00169 |

* H_D_, haplotype diversity (Nei 1987, equation 8.4); π, nucleotide diversity (Nei 1987, equation 10.5)

Nei M (1987) *Molecular evolutionary genetics*. Columbia University Press, New York, USA.
