# Supplementary material for: Discrete phenotypes are not underpinned by genome-wide genetic differentiation in the squat lobster Munida gregaria (Crustacea: Decapoda: Munididae): a multi-marker study covering the Patagonian shelf
Source: BMC Evol Biol. 2016 Dec 1;16:258. doi: 10.1186/s12862-016-0836-4 (PMC5131467; doi:10.1186/s12862-016-0836-4)
Supplement: Additional file 3: Table S2. — Diversity indices of nine microsatellite loci for the two ecotypes. Reported are number of alleles nA, fragment size range, observed heterozygosity H O, expected heterozygosity H E and allelic richness Ar. Significant deviation from Hardy-Weinberg equilibruim (P < 0.05, based on 10,000 permutations) after Bonferroni correction were labeled in bold. (DOCX 19 kb) [file 12862_2016_836_MOESM3_ESM.docx]

Table S2: Diversity indices of nine microsatellite loci for the two ecotypes. Reported are number of alleles nA, fragment size range, observed heterozygosity *H_O_*, expected heterozygosity *H_E_* and allelic richness *Ar*. Significant deviation from Hardy-Weinberg equilibruim (*P* < 0.05, based on 10,000 permutations) after Bonferroni correction were labeled in bold.

|  | nA | Size range | *subrugosa*_NCP | | *subrugosa*_TdF | | *subrugosa*_Total | |
| --- | --- | --- | --- | --- | --- | --- | --- | --- |
|  |  |  | *H_O_/ H_E_* | *Ar* | *H_O_/ H_E_* | *Ar* | *H_O_/ H_E_* | *Ar* |
| mgr4 | 18 | 266-341 | 0.75/0.706 | 4.13 | 0.533/0.615 | 6.02 | 0.579/0.634 | 13.03 |
| mgr8 | 21 | 284-364 | 0.916/0.793 | 7.53 | 0.844/0.838 | 6.93 | 0.860/0.832 | 13.68 |
| mgr46 | 6 | 148-172 | 0.166/0.159 | 2.79 | 0.133/0.208 | 2.43 | 0.140/0.198 | 4.39 |
| mgr52 | 11 | 160-180 | 0.833/0.753 | 5.32 | 0.666/0.753 | 5.07 | 0.702/0.747 | 7.93 |
| mgr60 | 38 | 170-274 | 1/0.913 | 11.91 | **0.755/0.895** | 11.36 | 0.807/0.897 | 29.44 |
| mgr62 | 7 | 155-167 | 0.5/0.489 | 3.38 | 0.377/0.406 | 3.25 | 0.404/0.424 | 5.24 |
| mgr81 | 9 | 296-322 | 0.666/0.594 | 3.53 | 0.644/0.611 | 4.05 | 0.649/0.607 | 7.09 |
| mgr90 | 9 | 232-256 | 0.75/0.807 | 6.16 | 0.8/0.822 | 6.22 | **0.789/0.832** | 7.88 |
| mgr120 | 15 | 249-318 | 0.333/0.307 | 4.12 | 0.355/0.339 | 3.95 | 0.351/0.332 | 9.59 |

|  | *gregaria*. s. str._FM | | *gregaria*. s. str.__NCP | | *gregaria*. s. str.__TdF | | *gregaria*. s. str.__Total | |
| --- | --- | --- | --- | --- | --- | --- | --- | --- |
|  | *H_O_/ H_E_* | *Ar* | *H_O_/ H_E_* | *Ar* | *H_O_/ H_E_* | *Ar* | *H_O_/ H_E_* | *Ar* |
| mgr4 | 0.720/0.768 | 6.98 | 0.637/0.676 | 5.45 | 0.7/0.805 | 7.00 | 0.689/0.737 | 13.54 |
| mgr8 | 0.838/0.816 | 6.62 | 0.810/0.829 | 7.46 | 1/0.773 | 6.77 | 0.839/0.816 | 13.70 |
| mgr46 | 0.129/0.161 | 2.16 | 0.206/0.249 | 2.67 | monomorphic | 1.98 | 0149/0.184 | 3.68 |
| mgr52 | 0.763/0.735 | 5.12 | 0.689/0.762 | 5.07 | 0.7/0.7 | 4.81 | 0.733/0.744 | 7.62 |
| mgr60 | 0.752/0.870 | 11.17 | 0.896/0.887 | 11.08 | 0.7/0.905 | 11.60 | 0.801/0.880 | 29.43 |
| mgr62 | 0.516/0.483 | 3.17 | 0.534/0.589 | 3.53 | 0.8/0.573 | 2.83 | 0.540/0.536 | 5.47 |
| mgr81 | 0.677/0.627 | 4.03 | 0.620/0.626 | 4.54 | 0.4/0.673 | 2.98 | 0.640/0.633 | 7.56 |
| mgr90 | **0.870/0.820** | 6.32 | **0.844/0.830** | 6.18 | 1/0.805 | 4.98 | **0.870/0.824** | 8.07 |
| mgr120 | 0.290/0.311 | 3.77 | 0.396/0.376 | 4.03 | 0.1/0.1 | 3.65 | 0.317/0.324 | 9.40 |
